# Characterization and in vitro assessment of three-dimensional extrusion Mg-Sr codoped SiO_2_-complexed porous microhydroxyapatite whisker scaffolds for biomedical engineering

**DOI:** 10.1186/s12938-021-00953-w

**Published:** 2021-11-24

**Authors:** Chengyong Li, Tingting Yan, Zhenkai Lou, Zhimin Jiang, Zhi Shi, Qinghua Chen, Zhiqiang Gong, Bing Wang

**Affiliations:** 1 Department of Orthopedics, First Affiliated Hospital of Kunming Medical University, Kunming Medical University, Kunming, 650032 China; 2 Faculty of Materials Science and Engineering, Kunming University of Science and Technology, Kunming, 650093 China

**Keywords:** Micron hydroxyapatite whiskers, Extrusion molding, Porous ceramic scaffold, Bone tissue engineering

## Abstract

**Background:**

Large bone defects have always been a great challenge for orthopedic surgeons. The use of a good bone substitute obtained by bone tissue engineering (BTE) may be an effective treatment method. Artificial hydroxyapatite, a commonly used bone defect filler, is the main inorganic component of bones. Because of its high brittleness, fragility, and lack of osteogenic active elements, its application is limited. Therefore, its fragility should be reduced, its osteogenic activity should be improved, and a more suitable scaffold should be constructed.

**Methods:**

In this study, a microhydroxyapatite whisker (mHAw) was developed, which was doped with the essential trace active elements Mg^2+^ and Sr^2+^ through a low-temperature sintering technique. After being formulated into a slurry, a bionic porous scaffold was manufactured by extrusion molding and freeze drying, and then SiO_2_ was used to improve the mechanical properties of the scaffold. The hydrophilicity, pore size, surface morphology, surface roughness, mechanical properties, and release rate of the osteogenic elements of the prepared scaffold were detected and analyzed. In in vitro experiments, Sprague–Dawley (SD) rat bone marrow mesenchymal stem cells (rBMSCs) were cultured on the scaffold to evaluate cytotoxicity, cell proliferation, spreading, and osteogenic differentiation.

**Results:**

Four types of scaffolds were obtained: mHAw-SiO_2_ (SHA), Mg-doped mHAw-SiO_2_ (SMHA), Sr-doped mHAw-SiO_2_ (SSHA), and Mg-Sr codoped mHAw-SiO_2_ (SMSHA). SHA was the most hydrophilic (WCA 5°), while SMHA was the least (WCA 8°); SMHA had the smallest pore size (247.40 ± 23.66 μm), while SSHA had the largest (286.20 ± 19.04 μm); SHA had the smallest Young's modulus (122.43 ± 28.79 MPa), while SSHA had the largest (188.44 ± 47.89 MPa); and SHA had the smallest compressive strength (1.72 ± 0.29 MPa), while SMHA had the largest (2.47 ± 0.25 MPa). The osteogenic active elements Si, Mg, and Sr were evenly distributed and could be sustainably released from the scaffolds. None of the scaffolds had cytotoxicity. SMSHA had the highest supporting cell proliferation and spreading rate, and its ability to promote osteogenic differentiation of rBMSCs was also the strongest.

**Conclusions:**

These composite porous scaffolds not only have acceptable physical and chemical properties suitable for BTE but also have higher osteogenic bioactivity and can possibly serve as potential bone repair materials.

**Supplementary Information:**

The online version contains supplementary material available at 10.1186/s12938-021-00953-w.

## Background

Bones are the main supports and structures for movement in the human body and can provide protective spaces for the brain, heart, lungs, liver, and other organs. Bones also possess a robust regenerative capacity and can regenerate completely under the appropriate conditions after they are broken or slightly injured. Bone healing consists of three continuous and partially overlapping processes [1]. In the first stage, immunoregulation, stem cell recruitment, and chondrogenesis occur. In the second stage, cartilage is absorbed, and new bone is formed under the combined action of osteoclasts and osteoblasts. In the third stage, the newly formed remodeled bone returns to normal, and the repair is complete. However, when a bone defect is too large to heal itself, it is called a large-size or critical-size bone defect, and the repair process cannot be spontaneously completed.

Large bone defects are always caused by severe trauma, tumor removal, infection, or congenital malformation, and reconstruction of these defect sites is a major challenge for orthopedic surgeons and patients [2, 3]. Currently, the main treatment measures for these large bone defects include autogenous bone grafts, allografts, and artificial bone substitutes [3–5]. However, the above treatment methods have specific deficiencies during the treatment process. Autografts, as the gold standard, have complications, including an increased surgical site, limited bone mass, bleeding, and additional pain. Allografts frequently pose risks, including immunological rejection and pathophoresis from the donor. Hopefully, bone tissue engineering (BTE) can provide more choices for patients with bone defects, although there is still much to be done to approach or achieve the therapeutic effects of autologous bone. Notably, the application of single materials will always present problems, such as poor mechanical properties, poor osteogenic properties, tumorigenicity, and mismatch between the degradation rate and the rate of new bone formation [5, 6].

Over the past few years, BTE has been used to promote bone regeneration, as substantial efforts and confidence have been placed in scientific research. With the development of new technologies, different types of scaffolds have been established and applied in bone repair, but only a few scaffolds have shown satisfactory results [5, 7, 8]. Long bones are mostly composed of an outer layer of cortical bone and an inner layer of cancellous bone, and the proportion and distribution of cancellous or cortical bone of irregular bones, such as vertebrae and skull, vary according to their functions. The major organic component of natural bone is type I collagen (COL1), and the major inorganic component is hydroxyapatite (HA: Ca_10_(PO_4_)_6_(OH)_2_) [9], which also contains necessary elements, such as Mg^2+^, Si^4+^, and Sr^2+^, and other metal ions [5, 10–13].

As the main inorganic component of natural bone, HA has excellent biocompatibility, good cell adhesion, and good osteoconductivity [9, 14–16] and can be divided into macroscopic, micron, and nanoscale sizes. Research in recent years has confirmed that, compared with scaffolds based on HA particles, scaffolds based on micron-sized HA during bone repair have better hierarchical porous structures and enhanced mechanical properties as well as improved biological activity and biological responses [18–20]. The trace elements Mg^2+^, Si^4+^, and Sr^2+^ in bone also have important osteogenic and vascular functions. Mg has good osteoconductivity and osteoinductivity, promotes vasodilation, sprawling, and new blood vessel formation, increases blood perfusion, and has good tissue affinity. It participates in early osteogenic differentiation, midterm new bone formation, and late bone remodeling [17–19]. Mg and Sr also have good biological safety in tissues and the blood and certain antibacterial properties (including resistance to methicillin-resistant *Staphylococcus aureus*) [20–22]. However, its rapid degradation rate limits its applications in orthopedics [23]. Si is nontoxic or has only very low toxicity and shows good biocompatibility, degradability, and biological excretion [24]. Si^4+^ can recruit bone marrow mesenchymal stem cells (BMSCs) and promote their osteogenic differentiation in the early stage, improve the adhesion and proliferation of osteoblasts, and promote the formation and structural stabilization of COL1. Si^4+^ can also promote the precipitation and mineralization of the bone matrix in the midterm phase of bone repair. Moreover, angiopoietin-2 is upregulated through the cell signaling pathway to regulate blood vessel formation [5, 25–27]. Sr has outstanding antiosteoporosis performance through the inhibition of the differentiation and activity of osteoclasts and has certain osteogenic and strong angiogenic functions, as well as antibacterial properties [28–33]. In addition, when multiple ions work together, their bone repair and antibacterial effects are better [9, 12, 31, 34, 35]. An ideal bone repair material should be biocompatible, biodegradable, and nontoxic, has suitable physical properties, and shows good osteogenic and angiogenic abilities [4, 9, 34].

Inspired by the composition and porous structure of natural bone and the osteogenic function of mHAws, including magnesium, strontium, and silica mentioned above, this study aimed to develop a biomimetic porous scaffold as a bone filling material with acceptable mechanical strength, a structure similar to natural bone, and good osteogenesis-promoting effects. Its mechanical strength was closer to that of natural bone enough to support local soft tissues. It had a rough surface microenvironment suitable for cell growth, similar to the porous structure of cancellous bone, and achieved the local sustained release of the osteogenic active elements silicon, magnesium, and strontium. In vitro experiments were performed to verify its cell proliferation and osteogenic differentiation ability.

## Results

### Fabrication and characterization of the scaffolds

**Fig. 1:**
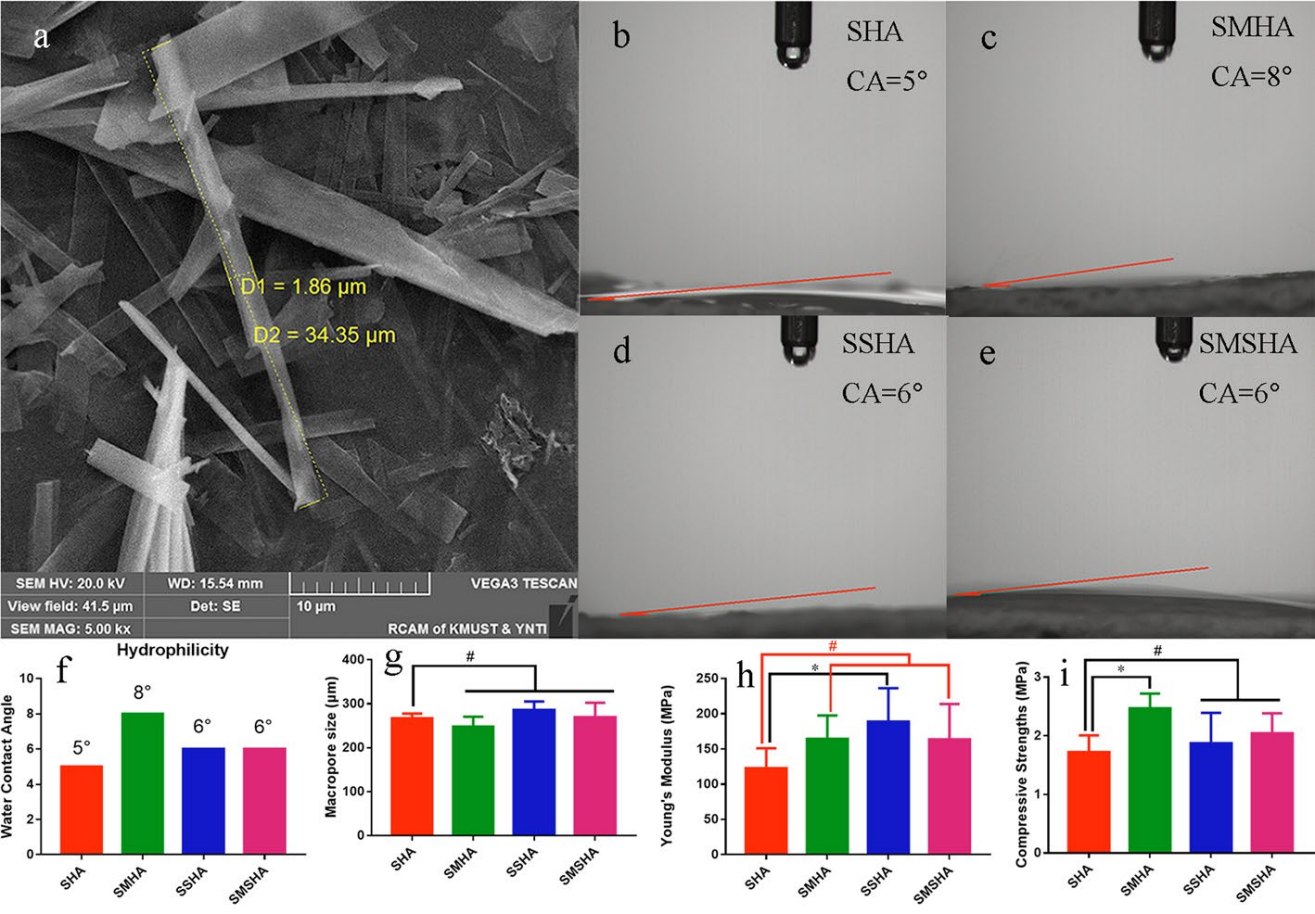
Morphologies of the microhydroxyapatite whiskers observed by SEM (**a**). Hydrophilic images of SHA, SMHA, SSHA, and SMSHA (**b**–**e**), respectively. Water contact angles of SHA, SMHA, SSHA, and SMSHA (**f**). Pore sizes of the four scaffolds (**g**). Young’s moduli of the four scaffolds (**h**). The compressive strengths of four scaffolds (**i**). (n = 5, * *P* < 0.05, # *P* > 0.05 compared with SHA)

Pure mHAw-, Mg-doped mHAw-, Sr-doped mHAw-, and Mg-Sr codoped mHAw were successfully synthesized. Scanning electron microscopy (SEM) observations and analysis showed that the lengths of the prefabricated mHAws ranged from a few microns to tens of microns, while the mHAws were several microns in diameter (Fig. 1a). The SHA, SMHA, SSHA, and SMSHA scaffolds were prepared according to the mentioned method, as shown in Fig. 9. Briefly, through low-temperature sintering, the doped mHAws were extruded into porous ceramics, complexed with silica to enhance their mechanical strength, and cut to the required size.

**Fig. 2:**
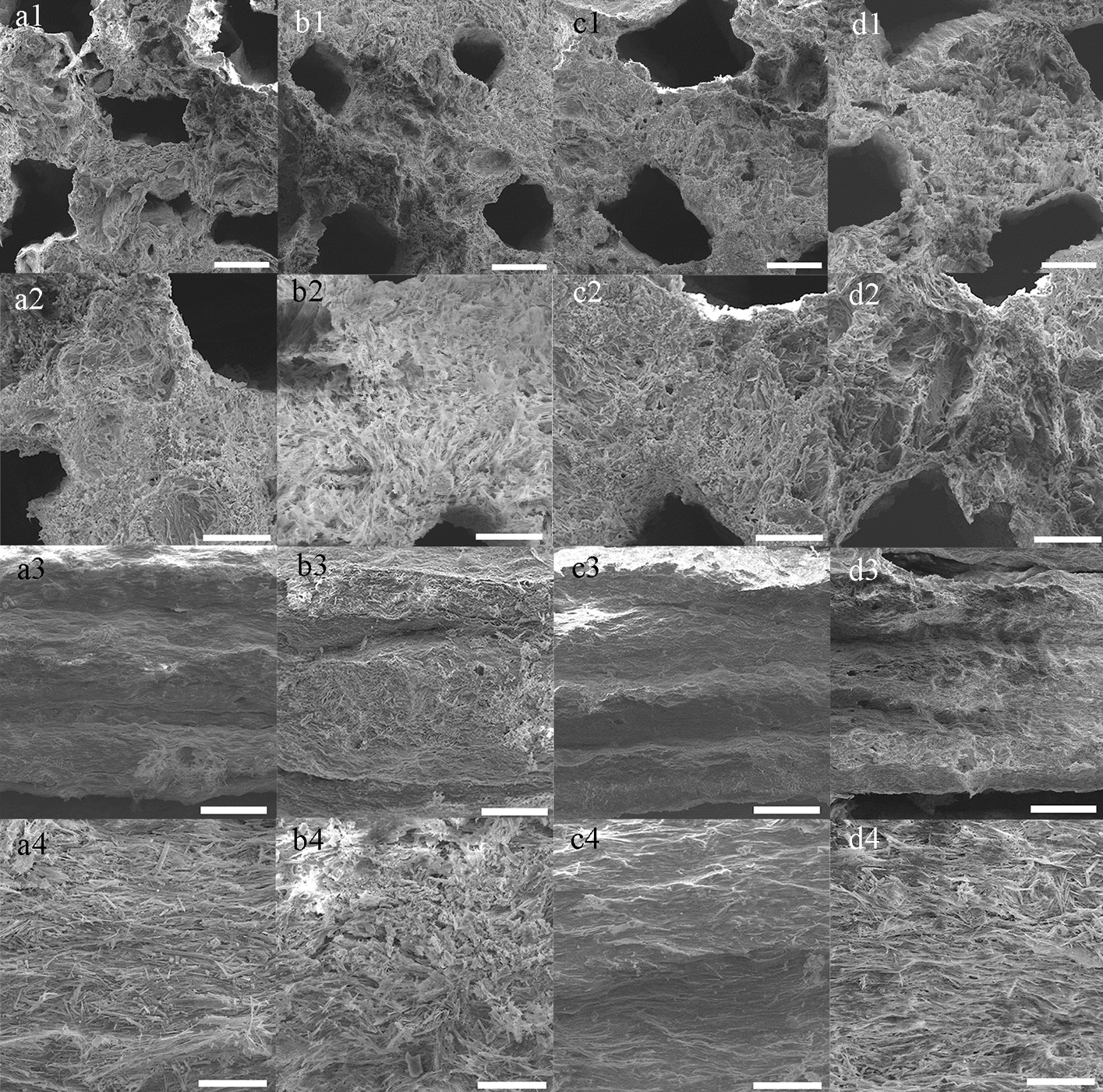
Surface morphology and porous structure analyses. The macroporous surfaces of SHA (**a1**–**a2**), SMHA (**b1**–**b2**), SSHA (**c1**–**c2**), and SMSHA (**d1**–**d2**). The microporous surfaces of SHA (**a3**–**a4**), SMHA (**b3**–**b4**), SSHA (**c3**–**c4**), and SMSHA (**d3**–**d4**)

## Hydrophilicity properties

As shown in Fig. 1b–f, the WCAs of the SHA, SMHA, SSHA, and SMSHA scaffolds were 5°, 8°, 6°, and 6° after measurement, respectively. The smaller the WCA, the better the hydrophilicity. The WCAs of the four scaffolds all reflected good hydrophilicity. SHA had the smallest WCA, SMHA had the largest WCA, and SSHA and SMSHA had the same WCA, indicating that the hydrophilicity of the scaffolds will be affected after doping with different ions. These biomaterials had good hydrophilicity, which is conducive to the attachment and growth of cells [36, 37]. As SMHA had the largest WCA, SHA had the smallest WCA, and SSHA and SMSHA (both doped with Sr) had smaller WCAs than SMHA, these results indicate that Sr is more hydrophilic than Mg.

### Pore sizes of the scaffold

Since a significant feature of natural bone is its porous structure, the design of the porous structure in ceramic bone scaffolds can provide favorable conditions for bone regeneration. The average pore sizes of SHA, SMHA, SSHA, and SMSHA were 267.20 ± 10.66 μm, 247.40 ± 23.66 μm, 286.20 ± 19.04 μm, and 269.40 ± 33.00 μm, respectively (Fig. 1g). Thus, the pore sizes of the four scaffolds were similar.

### Mechanical properties

Compared with natural bone, most of the scaffolds that are developed for bone regeneration have insufficient mechanical properties. Therefore, it is necessary to develop a preparation process to improve the mechanical properties of scaffolds, and material methods and structural improvements have been used to achieve this goal. To reduce the risks of the high brittleness and fragility associated with hydroxyapatite, the prepared pure mHAw, Mg-doped mHAw, Sr-doped mHAw, and Mg-Sr codoped mHAw scaffolds were immersed in silica gel, and the mechanical properties of the scaffolds were found to be enhanced by SiO_2_ complexation. The Young’s moduli of SHA, SMHA, SSHA, and SMSHA were 122.43 ± 28.79 MPa, 164.56 ± 32.99 MPa, 188.44 ± 47.89 MPa, and 163.28 ± 50.55 MPa, respectively, as shown in Fig. 1h.

When developing a new bone tissue engineering material, in addition to other performance requirements for specific applications, the goal should be to prepare strong and stiff materials. HA is a weak bioceramic, so it cannot be used alone as the main load-bearing bone substitute in the human body. A high compressive strength can effectively support the surrounding tissues to prevent collapse [38], and the appropriate compressive strength can be adapted to the strength of natural bone. The compressive strengths of SHA, SMHA, SSHA, and SMSHA were 1.72 ± 0.29 MPa, 2.47 ± 0.25 MPa, 1.87 ± 0.52 MPa, and 2.04 ± 0.35 MPa, respectively (Fig. 1i), and both SMHA and SMSHA were in the range of cancellous bone [39].

### SEM observations and surface roughness measurement

Promising scaffolds should have a suitable microscopic morphology to allow sufficient cell contact for the stimulation of cell responses. SEM confirmed that the four scaffolds (SHA, SMHA, SSHA, and SMSHA) had rough surface morphologies, meeting the microscopic morphology required for osteogenic differentiation and the growth of BMSCs. The surface of the scaffold with macropores is defined as the macroporous surface of the scaffold (Fig. 2a1–a2, b1–b2, c1–c2, and d1–d2), and the surface without macropores is defined as the microporous surface of the scaffold (Fig. 2a3–a4, b3–b4, c3–c4, and d3–d4). A macroporous surface has lotus root-like pores, which facilitate blood penetration, blood vessel formation, and the other functions mentioned above. More importantly, it also provides a bionic microenvironment for the stimulation of BMSC bioactivity.

**Fig. 3:**
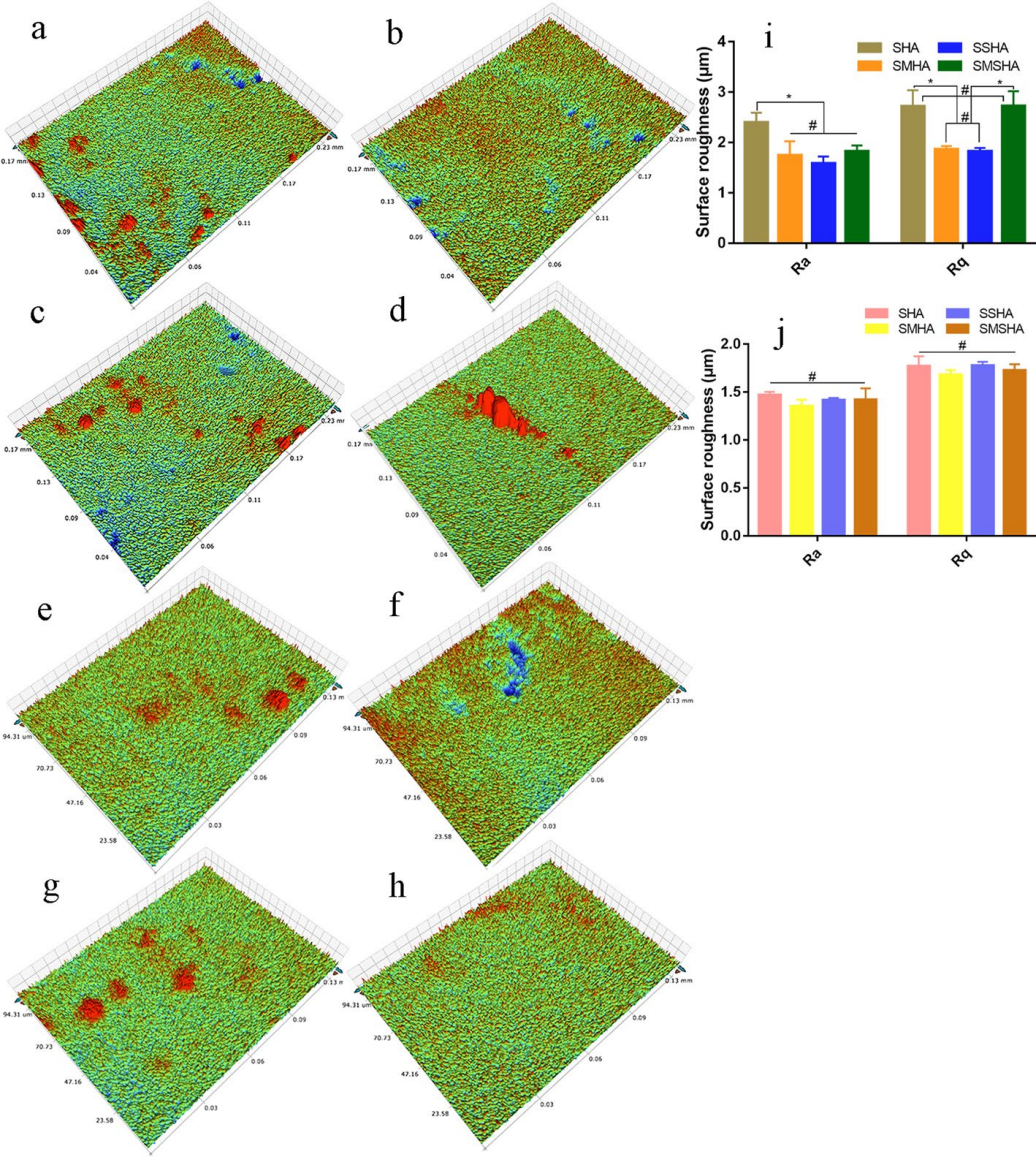
Optical profiler images of surface roughness. The surface roughness levels of the macroporous surfaces of SHA (**a**), SMHA (**b**), SSHA (**c**) and SMSHA (**d**); the surface roughness levels of the microporous surfaces of SHA (**e**), SMHA (**f**), SSHA (**g**) and SMSHA (**h**). Macroporous surface roughness parameters (**i**), microporous surface roughness parameters (**j**), *Ra* average roughness, *Rq* root mean square roughness, (n = 3, * P < 0.05, # P > 0.05)

Moreover, both the macroporous surface and the microporous surface are rough, which is conducive to the attachment of cells. The optical profiler analysis results showed that the surface roughness levels of the scaffolds were different. In terms of the surface roughness of the macroporous surface, SHA was the highest, followed by SMSHA. In terms of the microporous surface, the roughness levels of the four scaffolds were similar, as shown in Fig. 3.

**Fig. 4:**
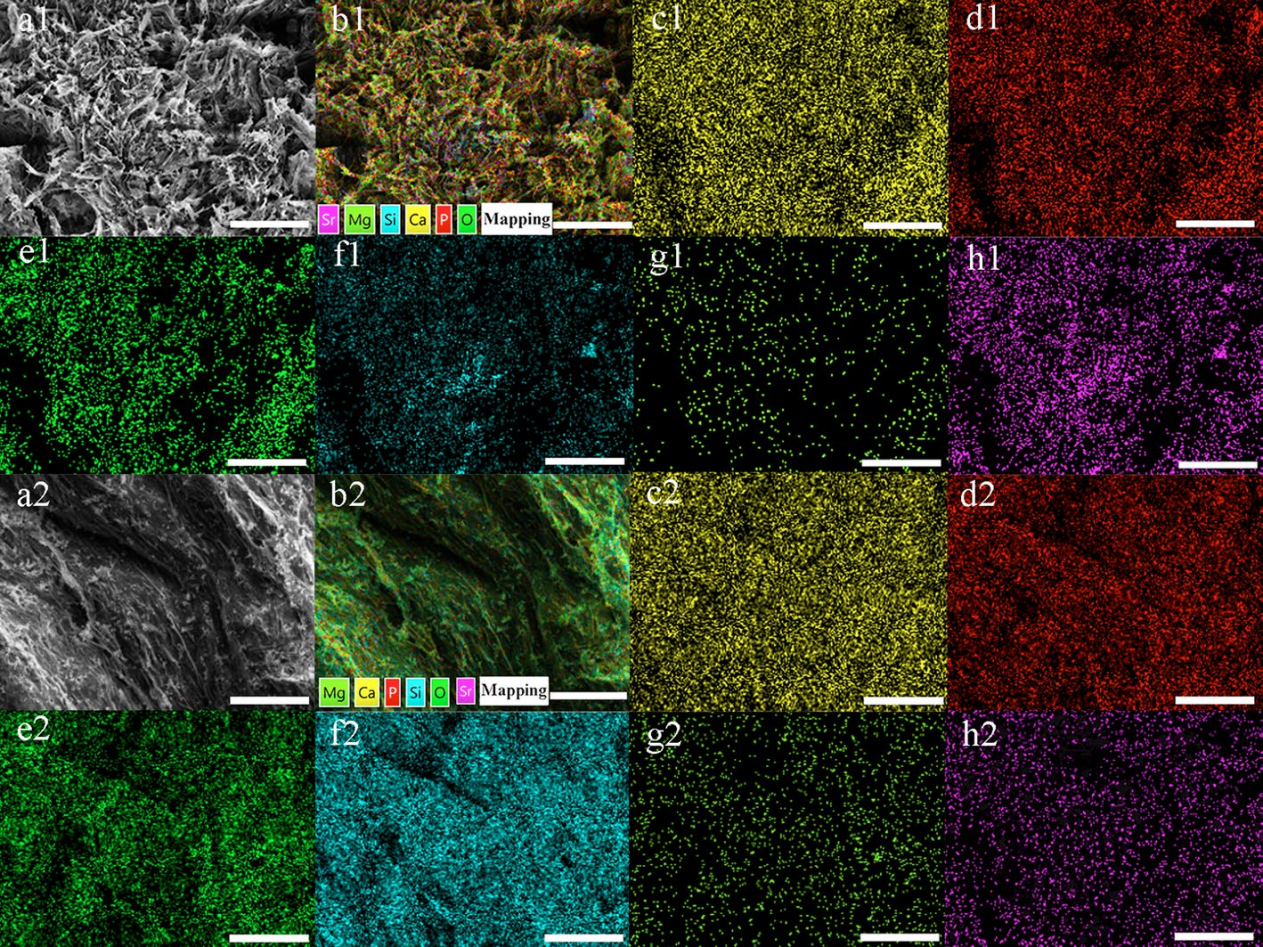
Elemental mapping images of SMSHA. SEM images (**a1**, **a2**). All elemental distribution images (**b1**, **b2**). Ca, P, O, Si, Mg, Sr distribution images (**c1**–**h1**, **c2**–**h2**), respectively. Macroporous surfaces (**a1**–**h1**) and microporous surfaces (**a2**–**h2**), respectively

**Fig. 5:**
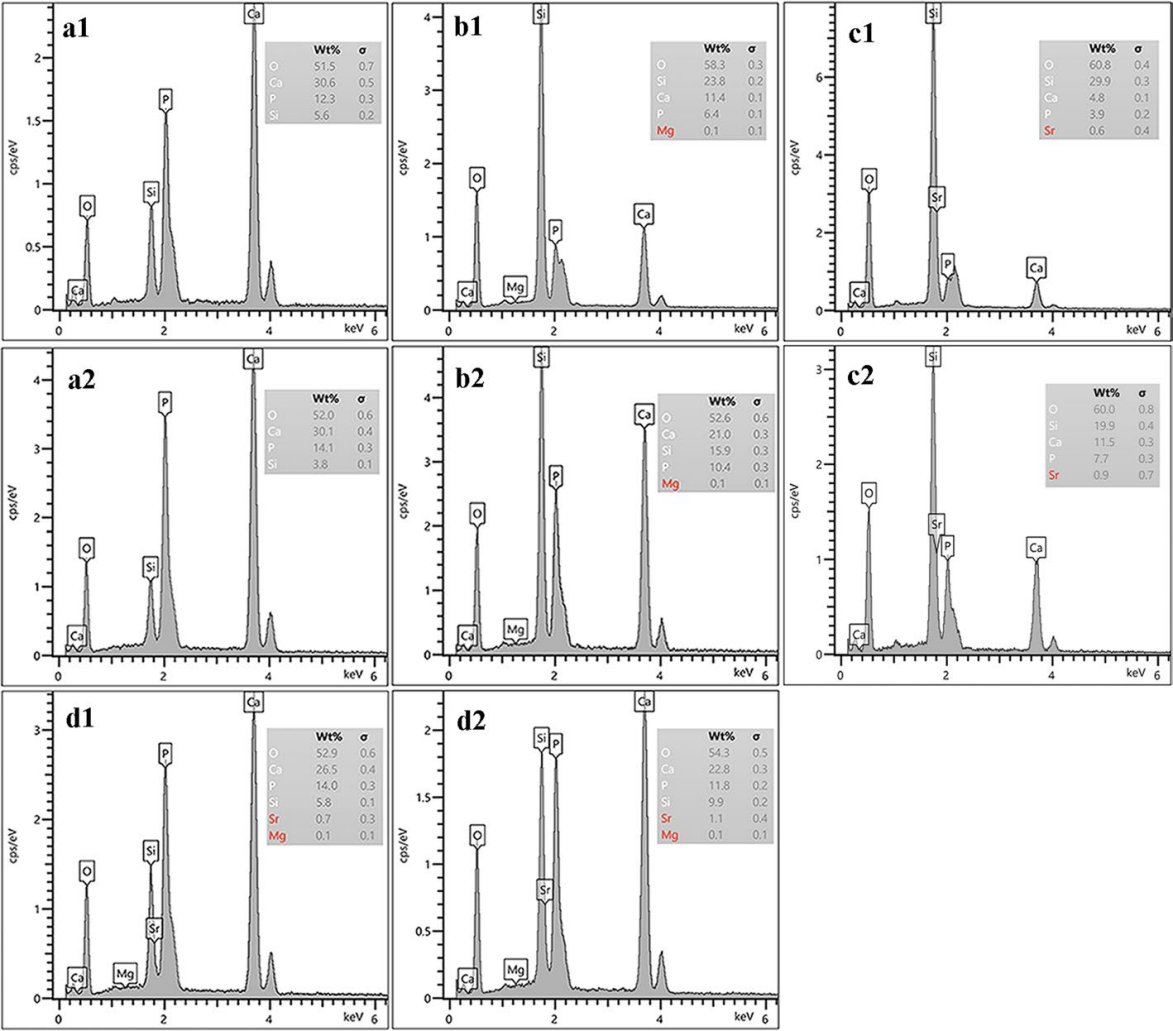
Macroporous surface EDS patterns of the prepared SHA, SMHA, SSHA, and SMSHA scaffolds (**a1**–**d1**). Microporous surface EDS patterns of the prepared SHA, SMHA, SSHA, and SMSHA scaffolds (**a2**–**d2**)

### Element distribution and content of the scaffolds

Except for silicon and oxygen, EDS element mapping showed that the distribution density of each element on the macroporous surface and the microporous surface was similar for each scaffold. Analysis showed that the main elements of SHA (Ca, P, O, and Si) (Additional file 1: Figure S1), SMHA (Ca, P, O, Si, and Mg) (Additional file 1: Figure S2), SSHA (Ca, P, O, Si, and Sr) (Additional file 1: Figure S3), and SMSHA (Ca, P, O, Si, Mg and Sr) (macroporous surface shown in Fig. 4b1–h1, microporous surface shown in Fig. 4b2–h2) were uniformly distributed on the corresponding scaffold. These results indicated that this doping method is effective and feasible.

Elemental content analysis showed that four main peaks of Ca, P, O, and Si could be detected for the four types of scaffolds on both macroporous surfaces (Fig. 5a1–d1) and microporous surfaces (Fig. 5a2–d2). Of the four scaffolds, Mg and Sr had different elements, SMHA had Mg peaks, SSHA had Sr peaks, and SMSHA had both Mg peaks and Sr peaks compared with SHA (Fig. 5a1–a2). However, the elemental contents of the macroporous surfaces and the microporous surfaces were slightly different, and the SMHA scaffold doped with Mg showed a lower content of Mg than the Sr content of the SSHA-doped Sr scaffold, which is related to the difficulty in replacing Ca^2+^ in mHAws with Mg^2+^ during the sintering process.

### Release of osteogenic active elements

**Fig. 6:**
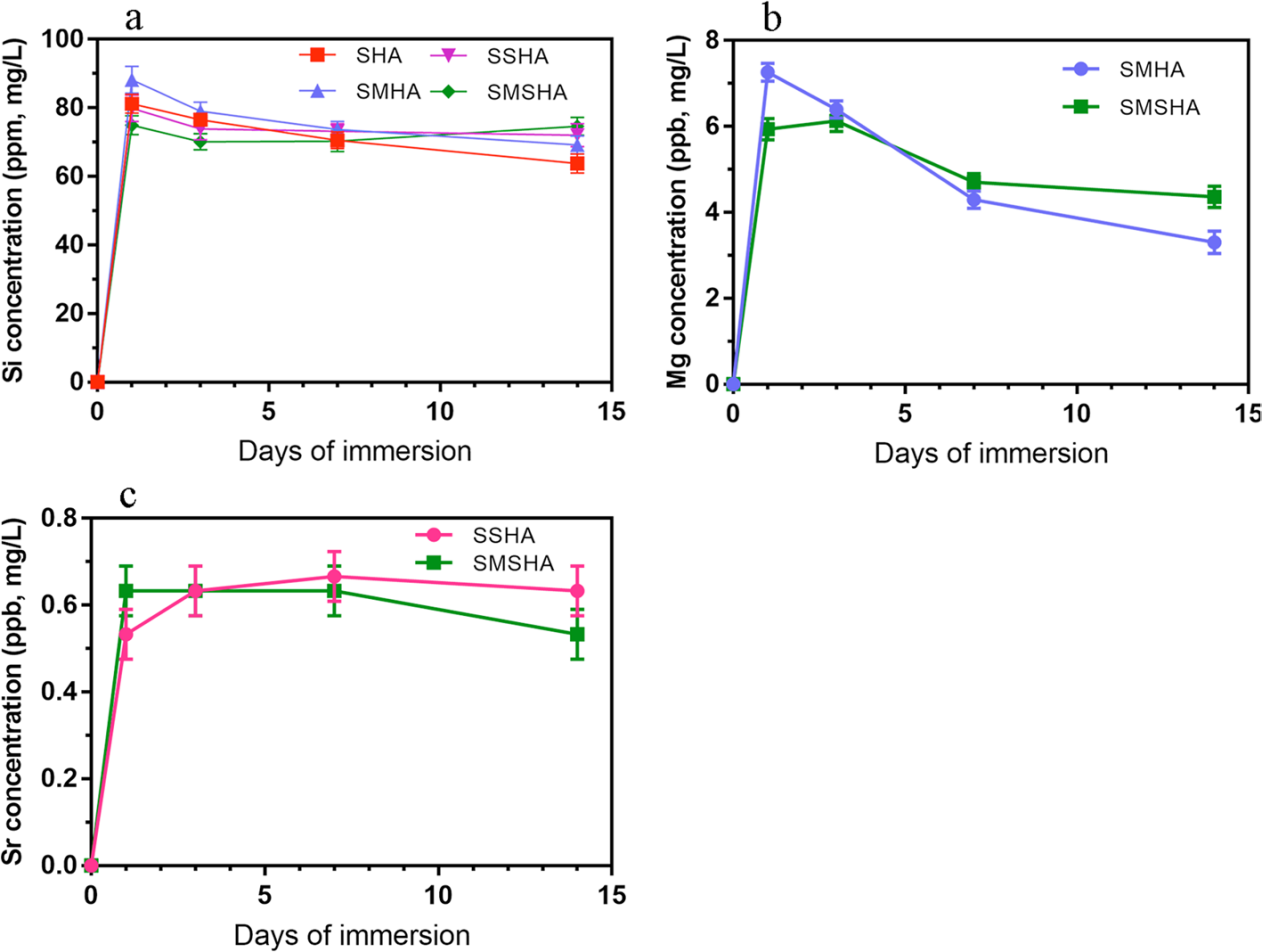
In vitro osteogenic active ion release profiles of SHA, SMHA, SSHA and SMSHA scaffolds. Si ion release profiles (**a**), Mg ion release profiles (**b**), Sr ion release profiles (**c**)

After the test, it was noted that each scaffold had its own unique ion release kinetics. The release rates of the four scaffolds' osteogenic active elements in PBS solution and the changes in the concentrations of Si, Mg, and Sr in PBS for different immersion times are shown in Fig. 6. Among them, the release of Si^4+^ was relatively stable, the release of Mg^2+^ showed a downward trend, and the release of Sr^2+^ was relatively stable.

### In vitro cell studies

#### Cell viability and proliferation

**Fig. 7:**
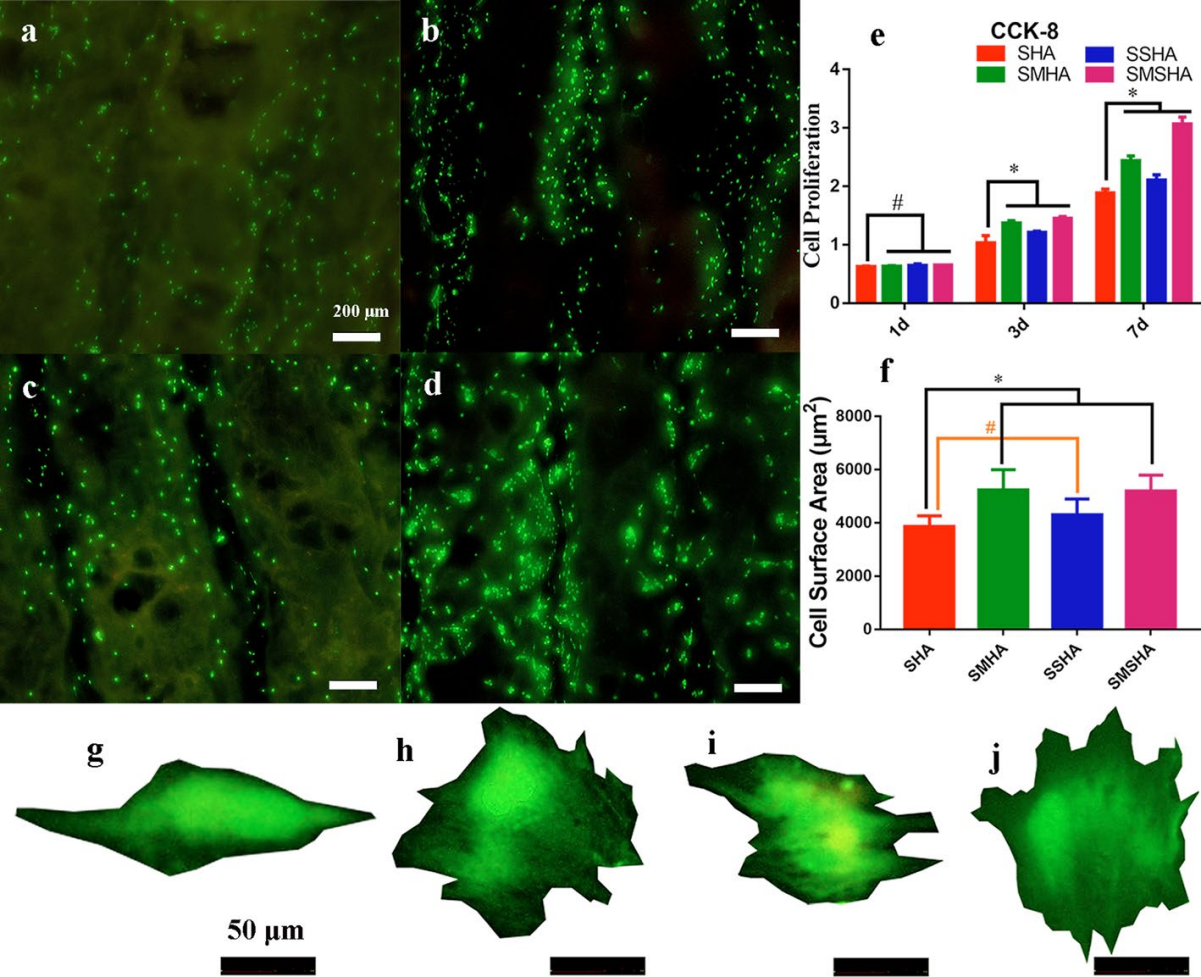
Live/dead staining to assess the viability of rBMSCs cultured on the SHA, SMHA, SSHA, and SMSHA scaffolds (**a**–**d**), respectively. The CCK-8 assay assessed the proliferation of rBMSCs (**e**) (n = 5, * *P* < 0.05, # *P* > 0.05 compared with SHA). The surface area (**f**) and typical cell morphology (**g**–**j**) of rBMSCs cocultured with SHA, SMHA, SSHA, and SMSHA for 3 days, respectively (n = 5, * P < 0.05, # P > 0.05 compared with SHA)

Cell live/dead staining confirmed that these scaffolds are beneficial to cell viability and growth. Living cells were stained green by calcein AM, and dead cells were stained red by EthD-1, as observed by upright fluorescence microscopy. Most of the cells on the scaffolds were green living cells, and the red-stained dead cells were almost invisible (Fig. 7a–d). The CCK-8 assay is a typical way to test the proliferation of cells. CCK-8 solution reacts with dehydrogenase in the mitochondria of living cells to produce yellow formazan, and the amount of formazan produced is proportional to the number of living cells. A microplate reader was used to measure the optical density (OD) values to evaluate the number of living cells, which indirectly reflects the cytotoxicity of the scaffolds. The rBMSCs cultured on the four scaffolds had good viability, as shown in the experimental results in Fig. 7e. The results of the live/dead staining and CCK-8 assays confirmed both that these four scaffolds had good biocompatibility and that their biosafety was favorable for the proliferation and spread of rBMSCs.

#### Surface area and morphological shape of rBMSCs

After culturing for 3 and 7 days, observation of the morphological shape of the rBMSCs with an upright fluorescence microscope verified that imaging cells cocultured with the scaffolds may be a simple way to assess the area of cell spreading organization and the cytoskeletal structure [40–42]. The shapes of the rBMSCs cocultured with the four types of scaffolds all showed different spreading organization areas (Fig. 7f–j). The analysis showed that SMHA and SMSHA were more conducive to an increase in cell spreading organization, which may be related to doping with Mg.

**Fig. 8:**
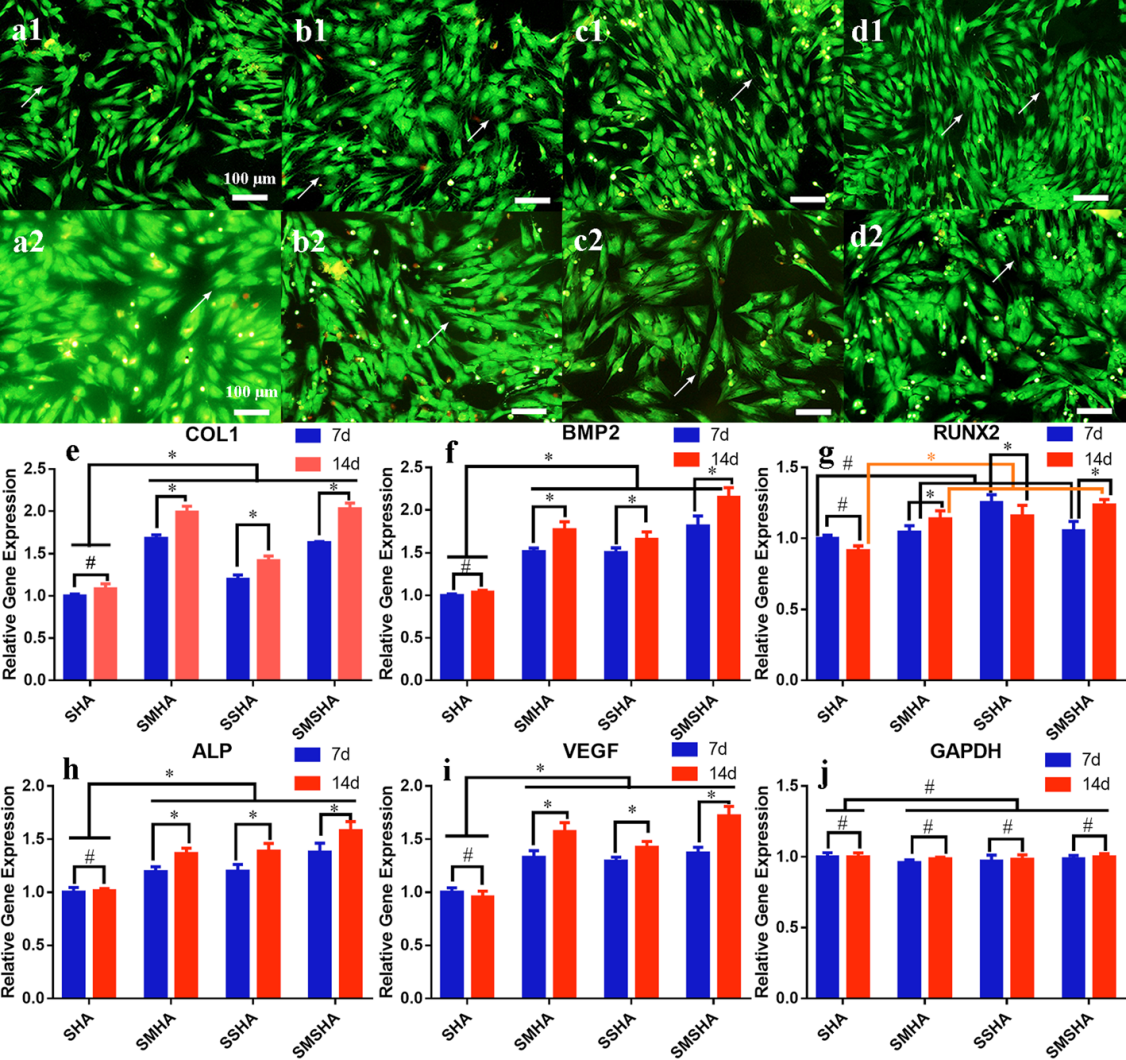
Morphology of the rBMSCs cocultured with the four scaffolds and fold changes in osteogenic-related gene expression in rBMSCs cultured on the four scaffolds. Morphologies of the rBMSCs cocultured with SHA, SMHA, SSHA, and SMSHA for 3 days (**a1**–**d1**) and 7 days (**a2**–**d2**) (10 ×), respectively; arrows indicate the filopodia. The changes in expression of COL1, BMP2, Runx2, ALP, and VEGF in rBMSCs cultured on the four scaffolds (**e**–**i**) compared with GAPDH (**j**) (n = 3, * *P* < 0.05, # *P* > 0.05 compared with SHA or 14 d compared with 7 d of each scaffold)

Bone regeneration in the defect site requires the proliferation and differentiation of the surrounding BMSCs to spread and cross, and cell spreading is driven by filopodia, pseudopods, and the cytoskeleton in a suitable physical and chemical environment. Morphological shapes, filopodia, and pseudopods were observed on rBMSCs cocultured with the four scaffolds and the rBMSCs cultivated with SMHA or SMSHA had a polygonal osteoblast-like shape and the largest numbers of filopodia and pseudopods (Fig. 8a1–d2).

#### Scaffolds induce the expression of genes related to bone formation in rBMSCs

Next, the osteogenic abilities of these scaffolds were further verified. After 7 and 14 days of culture on the scaffolds, the expression levels of rBMSC bone-specific genes, including COL1 (the main organic component of bone extracellular matrix), BMP2 (a factor that strongly promotes bone regeneration), Runx2 (an osteoblastic transcript factor that guides BMSCs to differentiate into osteoblasts), and ALP (a biochemical marker of early osteogenesis), were determined along with the angiogenic gene VEGF (a growth factor to enhance vascularization for tissue repair) and the housekeeping gene GAPDH (a constantly expressed gene) and were evaluated by RT–qPCR (the primer sequences are shown in Table 1). Compared with the SHA scaffold, the rBMSCs cultured on the SMHA, SSHA, and SMSHA scaffolds showed higher expression levels of COL1, BMP2, Runx2, ALP, and VEGF on the 7th and 14th days, and the SMSHA scaffold displayed the highest expression levels (Fig. 8e–i). The gene expression profiles of COL1, ALP, Runx2, and VEGF indicated good osteoblast proliferation and maturation.

**Table 1:** Primer sequences for RT–qPCR

Gene	Forward (5’-3’)	Reverse (3’-5’)
COL1	AAGAAGACATCCCTGAAG	AGATACAGATCAAGCATACA
BMP2	CATCACGAAGAAGCCATC	TCATCAGTAGGGACAGAAC
Runx2	AATGCCTCTGCTGTTATG	TTGTGAAGACCGTTATGG
ALP	TGATGCTCAGGACAGGAT	GGACCATAAGCGAGTTTCT
VEGF	CAGCATAGCAGATGTGAATG	TTCTCCGCTCTGAACAAG
GAPDH	CCTGCACCACCAACTGCTTA	GGCCATCCACAGTCTTCTGAG

## Discussion

The design of new bone filling materials for BTE needs to consider hydrophilicity, cytotoxicity, morphology, mechanical properties, osteogenic activity, and related factors. Composite bone repair materials based on hydroxyapatite have been a mainstream research topic in recent years, but their applications have been limited due to the inherent defects of hydroxyapatite. Based on the needs of bone tissue engineering and the goal of improving HA performance, active osteogenic elements are incorporated to improve the bone repair ability of the material. Micron hydroxyapatite whiskers have been researched and synthesized, and a porous scaffold has been successfully fabricated. Micron-sized HA may have better biological activity, and it can be increased to produce a biological scaffold with a porous structure [9]. These scaffolds have excellent hydrophilicity, and studies have revealed that the hydrophilic surface of biological materials can regulate the adsorption of fibronectin and fibrinogen and that these materials have a greater potential to promote the differentiation of macrophages into the anti-inflammatory phenotype [43, 44]. Notably, the doped Mg^2+^ and the hydrophilic surface of a material have some of the same cell signaling pathways (PI3K and NF-κB) in terms of exerting anti-inflammatory effects and assisting in osteogenesis [44–46].

Dense cortical bone has very few pores, spongy cancellous bone has many pores, and natural bone structure bionic materials have better application advantages in BTE [47]. These characteristics are closely related to the morphology and surface roughness of the scaffold. The pore sizes and surface roughness of the scaffold must match the requirements of bone regeneration to facilitate the attachment, migration, proliferation, and osteogenic differentiation of BMSCs and provide space for the deposition of calcium and phosphorus while promoting the formation and growth of blood vessels to provide nutrients and for the transportation of metabolic waste [4, 48–50]. The ideal pore size range for new bone regeneration is 150–350 μm [4]. mHAw-based scaffolds show roughness and have micropores, and the porous scaffold extruded by this die has more abundant pores with a suitable pore diameter, which is beneficial to the additive manufacturing of BTE. The effect of surface roughness, which results in a larger surface area for cell attachment, is an easy-to-customize and cost-effective factor that can affect cell behavior. The surface roughness of each scaffold is in the range of Ra ~ 0.7–3.1 μm, which facilitates the osteogenic differentiation of MSCs [51]. Therefore, the pore diameters and rough surface morphology of mHAw-based scaffolds may meet the needs of BTE.

The excellent mechanical properties of porous scaffolds can provide sufficient physical support and biochemical stimulation to facilitate bone formation [52]. The elastic modulus is an important index to measure the stiffness of a material, and Young’s modulus is the most important and characteristic mechanical property of elastic materials. The compressive strength levels of natural bone are 2–12 MPa for cancellous bone and 100–230 MPa for cortical bone [39]. The modulus of elasticity of cortical bone is 5–27 GPa and that of cancellous bone is 0.76–20 GPa [53]. In this study, silica was complexed with the scaffolds to enhance their mechanical properties, avoid the low strength of HA, and make the elastic moduli of the four scaffolds more similar to that of cancellous bone to support local soft tissues and demonstrated the good processing performance of mHAw. In addition, silicon has good osteogenic activity, which can provide a large amount of Si^4+^ during the degradation process of these scaffolds in bone defect sites. All scaffolds realized the sustained and stable release of Si^4+^, and the Si^4+^ release kinetics of the four scaffolds are similar.

HA chemically modified by ionic substitution has been demonstrated to be more advantageous than HA alone in accelerating bone regeneration and promoting the reabsorption of cell-mediated ceramic scaffolds [12, 54, 55]. Although SMHA and SMSHA scaffolds had difficulty doping Mg^2+^ and had less magnesium content, the release rates of Mg^2+^ were higher, and their abilities to induce the proliferation and osteogenic differentiation of BMSCs were also stronger. The live/dead, CCK-8, rBMSC area, and morphology results confirmed that SMSHA facilitated rBMSC attachment, proliferation, and spreading to a greater extent than the SHA, SMHA, and SSHA scaffolds, suggesting that SMSHA can release Si^4+^, Mg^2+^, and Sr^2+^, three active elements, to synergistically exert biological activity [56]. The larger the surface area of the BMSCs is, the greater the possibility of osteogenesis. Additionally, the smaller the area of cell spreading organization is, the greater the tendency of BMSCs to undergo apoptosis [41]. High-efficiency Mg^2+^ release has a significant role in promoting the osteogenic activity of rBMSCs [42]. Although the strontium contents in SSHA and SMSHA were high, its release rates were low. The release kinetics of Mg^2+^ and Sr^2+^ are variable, so there are differences in in vitro biological osteogenesis. Therefore, the cell proliferation and osteogenic activity of SSHA were lower those that of SMHA and SMSHA, which may be related to the ion release rate.

Bone-related genes are important factors that regulate bone regeneration. Compared with SHA, the expression levels of COL1, BMP2, ALP, VEGF, and Runx2 increased to varying degrees in rBMSCs cultured on the SMHA, SSHA, and SMSHA scaffolds, while GAPDH was constantly expressed, indicating that Mg^2+^ and Sr^2+^ can promote the expression of these factors and rBMSC osteogenic differentiation. These gene expression profiles may be stimulated by Mg^2+^, Sr^2+^, or the combination of Mg^2+^ and Sr^2+^ [8, 12, 57] on the basis of mHAws and silica. In the early stage of new bone formation, COL1 participates in bone mineralization to form osteoids, and in the later stage, COL1 aligns with HA crystals to form mature bone [8, 57]. Both Mg^2+^ and upregulated BMP2 can promote the osteogenic differentiation of BMSCs [8, 58]. VEGF plays an important stimulatory role in all stages of bone development and repair, including endochondral ossification and intramembranous ossification, and in bone, it is mainly derived from osteoblasts [59–61]. Runx2 can guide BMSCs to differentiate into osteoblasts and upregulate osteocalcin and osteopontin [62]. mHAws, silica, magnesium, and strontium have broad application prospects in bone regeneration. The use of Mg-Sr codoped mHAw scaffolds for bone repair may be a promising method to avoid the limitations of individual applications and can synergistically promote bone formation.

## Conclusions

Based on the strategy of low-temperature sintering and extrusion molding, four scaffolds were synthesized: SHA, SMHA, SSHA, and SMSHA, which were mainly composed of microhydroxyapatite whiskers doped with magnesium and strontium and complexed with silica to enhance the mechanical properties. Each scaffold had a high specific porous surface area, porosity, rough surface morphology, and sustained release of osteogenic active factors, promoting the adhesion and proliferation of SD rBMSCs. Compared with SHA, the SMHA, SSHA, and SMSHA scaffolds more effectively stimulated the osteogenic and angiogenic differentiation of rBMSCs in vitro. Therefore, the prepared scaffolds have strong potential application value in the field of biomedical bone tissue engineering. In further research, these four scaffolds will be transplanted into animals to verify their capacity to promote bone regeneration and new blood vessel formation in vivo.

## Materials and methods

### Scaffold fabrication and bone repair mechanism

The raw materials and their roles were calcium nitrate to provide calcium ions, diammonium hydrogen phosphate to provide phosphate, urea as a nitrogen source to form an alkaline environment, sorbitol as a template agent to guide whisker growth, and nitric acid as a catalyst. These raw materials were sequentially added to ultrapure water, and after being fully dissolved, they were made into a reaction solution, and configured with a ratios of Ca (or Ca + Mg, Ca + Sr, or Ca + Mg/Sr)/P of 1.67. The pH of each solution was adjusted to 2 ~ 3. The prepared solutions were heated in a water bath to 94 °C for 20 h to obtain the mHAws. The obtained mHAws were washed with distilled water 6 ~ 7 times, filtered with suction, and dried at 60 °C. Konjac glucomannan was then added to the hydroxyapatite whiskers at a ratio of 10%, an appropriate amount of ultrapure water was added and the solution was mixed well. (Konjac glucomannan was obtained by separated and removed starch, pigments, alkaloids, and sulfur dioxide from refined konjac powder. It plays the role of binder and thickener in the process of preparing whiskers, and could be completely removed in the sintering process.)

**Fig. 9:**
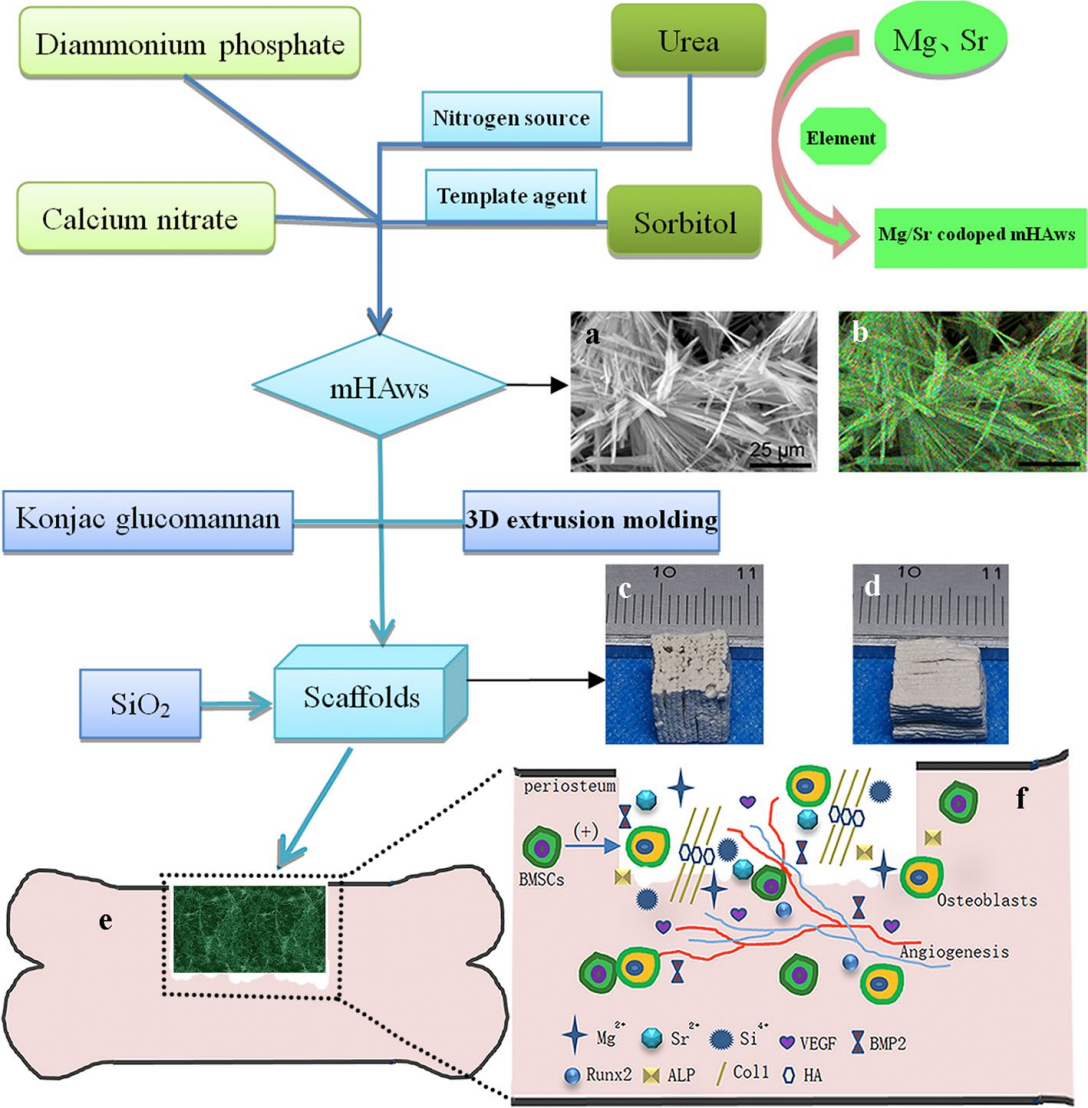
Fabrication of a porous hierarchical scaffold with Mg-Sr codoped SiO_2_-complexed microhydroxyapatite whiskers (mHAws). SEM micrograph of Mg-Sr codoped mHAws (**a**). Element mapping of Mg-Sr codoped mHAws (**b**). Prepared SMSHA scaffold (size 0.8 × 0.8 × 0.8 cm): the macroporous surface (**c**) and the microporous surface (**d**). Schematic diagram of scaffold filling bone defect (**e**) for BMSC osteogenic differentiation and bone regeneration (**f**)

The mixed slurry was extruded through a die to form a porous ceramic scaffold, which was freeze dried at minus 10 °C to remove the excess water from the scaffold. The dried scaffold was cut to the required size and placed in a muffle furnace for firing at 450 °C (sintering rate was 5 °C/min). The fired scaffold was immersed in liquid silica gel, removed after two hours, and dried, and the dried scaffold was placed in a muffle furnace again at 400 °C, and fired for an additional four hours to prepare a porous ceramic scaffold containing SiO_2_. In this way, the silica-complexed mHAw scaffold (SHA), silica-complexed Mg-doped mHAw scaffold (SMHA), silica-complexed Sr-doped mHAw scaffold (SSHA), and silica-complexed Mg-Sr codoped mHAw scaffold (SMSHA) were prepared, and the fabrication process is shown in Fig. 9. The mechanism by which SMSHA induces bone repair in bone defects is shown in Fig. 9e–f.

### Hydrophilicity tests

Orthopedic grafts need to have a certain degree of hydrophilicity. Testing the hydrophilicity is an indirect method to detect the potential biological application value of these materials. The hydrophilicity of each of the scaffolds was confirmed by measuring the water contact angle (WCA) of the scaffold surface using a water contact angle measurement instrument (CA100A, Shanghai, China). Briefly, 5 μl of ultrapure water was dropped onto the surface of the holder under ambient conditions, and then the contact angle was measured. All measurements were repeated 3 times on different parts of each scaffold.

### Pore size tests

A main characteristic of porous ceramic scaffolds for bone regeneration is their pores, which can perform specific functions. Five samples of each scaffold were randomly selected, and the pore size from the cross-sectional surface of each sample was randomly measured by scanning electron microscopy (SEM; TESCAN VEGA3, Czech Republic, Europe). Then, the average pore size in each of the corresponding scaffolds was calculated.

### Mechanical properties

The SHA, SMHA, SSHA, and SMSHA scaffolds were cut into 8 × 8 × 8 mm^3^ cubes, and then the mechanical properties of each scaffold were measured using a static and dynamic material testing machine (HY-0230, Shanghai, China) with a load of 240 N. Five samples in each group were tested at a speed of 1 mm/min, and load–displacement curves were obtained. According to ISO 844:2004, the load–displacement curves can be used to calculate the Young’s moduli and compressive strengths of the scaffolds.

### Morphology and structural characterization analysis

It is very important that the surface roughness and morphology of mHAw scaffolds mimic the porous structure of natural bone, and a rough surface morphology is conducive to the attachment, sprawling, proliferation, and osteogenic differentiation of BMSCs [50]. The mHAw scaffold surface morphology was verified by SEM, an optical profiler (Bruker Counter GT K 3D) was used to analyze the surface roughness parameters of the scaffolds, and the Ra (average roughness) and Rq (root mean square roughness) of all the scaffolds were calculated.

### Inductively coupled plasma optical emission spectrometry (ICP-OES) and energy dispersive spectroscopy (EDS) measurements

Scaffold cut into cubes (8 × 8 × 8 mm^3^) was placed in a glass container. According to the standards of GB/T 16,886.13–2001 and GB/T 16,886.12–2001, the mass volume ratio of scaffold and phosphate buffered saline (PBS, pH = 7.4) was 1 g:15 ml. After constant temperature (37 °C) and speed (70 rpm) shaking for 1, 3, 7, and 14 days, 5 ml of degradation solution was collected at each time point, and an equal volume of fresh PBS was added at the same time. ICP-OES (PE Avio 200) was used to determine the release rate of the osteogenic active element Si^4+^ in the four scaffolds, and inductively coupled plasma mass spectrometry (ICP-MS, iCAP Qc) was used to measure the ion release rates of Mg and Sr. A 10 kV FESEM (S-4800, HITACHI, Tokyo, Japan) equipped with energy dispersive spectroscopy (EDS) capabilities was used to scan the chemical morphology map and various elemental contents of the cross sections of each scaffold.

### In vitro cell experiments

#### Cell culture

Sprague–Dawley (SD) rats (120 g, 5–6 weeks old, purchased from the Department of Veterinary Medicine, Kunming Medical University) were euthanized and disinfected. Their femurs and tibias were removed from both sides, the epiphyses were cut at both ends, and the bone marrow cavities were flushed repeatedly with Dulbecco’s modified Eagle’s medium (DMEM; Gibco, USA). After centrifugation at 1000 rpm for 5 min, the cells were resuspended in DMEM supplemented with 1% penicillin/streptomycin and 10% fetal bovine serum (FBS; Gibco, USA) for cell culture. The cells were cultured in an incubator containing 5% carbon dioxide at 37 °C. Rat bone marrow-derived mesenchymal stem cells (rBMSCs) were obtained after 7 days of culture (the nonadherent cells were removed during the culture, and the cells were passaged when the fusion exceeded 80%), and passages 3 to 5 were used for cell experiments.

#### Cell viability and proliferation

After the rBMSCs (1 × 10^5^) were incubated on each scaffold (8 mm × 2 mm) for 3 days, the survival of the rBMSCs was determined by fluorescent staining; that is, rBMSCs were treated with a staining kit (Solarbio, China) containing calcein AM and EthD-1 for the live/dead staining assay. In this assay, dead cells were stained red, and living cells were stained green. To further evaluate the proliferation of rBMSCs cultured for 1 day, 3 days, and 7 days according to the aforementioned method, a sample of complete DMEM was removed at each time point, and 550 μl of DMEM containing 10% Cell Counting Kit-8 (CCK-8, Dojindo, Japan) solution was added to each microplate well. After incubation for 1–4 h under cell culture conditions, 5 replicate wells were set in a 96-well plate, and 100 μl of incubation solution was added to each well. The absorbance of each solution was measured with a microplate reader (Bio–Rad 680, USA) at a wavelength of 450 nm to evaluate the viability and proliferation ability of the rBMSCs on each scaffold.

#### Cell morphology and surface area

To analyze the effects of various scaffolds on cell morphology and surface area, rBMSCs cocultured with the four scaffolds were washed twice with PBS after coculture for 3 days. The cytoplasm of the rBMSCs was stained with calcein AM obtained from the live/dead viability kit and was then used to stain live cells. Fluorescence images were obtained with an upright fluorescence microscope (OLYMPUS, BX53F, Tokyo, Japan), the morphological shapes of the rBMSCs were observed, and then the typical cell surface areas were calculated using cellSens Standard software (OLYMPUS, Tokyo, Japan).

#### Osteogenic gene expression analysis

The effects of the scaffolds on the expression levels of rBMSC osteogenic genes were studied by real-time quantitative polymerase chain reaction (RT–qPCR). rBMSCs were seeded on each scaffold in duplicate at a density of 2 × 10^5^ cells per scaffold. After 7 or 14 days of culture, the cells on the scaffold were lysed with 1 ml of TRIzol (Invitrogen, USA) to isolate and obtain total RNA. Complementary DNA (cDNA) was synthesized using the PrimeScript First Strand cDNA Synthesis kit (Thermo Scientific, Lithuania) according to the manufacturer’s instructions. A real-time PCR kit (FastStart Universal SYBR^@^ Green Master, Roche, Germany) was used on a thermal cycler (Applied Biosystems, Australia) to analyze type I collagen (COL1), bone morphogenetic protein 2 (BMP2), Runt-related transcription Factor 2 (Runx2), alkaline phosphatase (ALP), and vascular endothelial growth factor (VEGF) cDNA. The housekeeping gene glyceraldehyde 3-phosphate dehydrogenase (GAPDH) was used to analyze the changes in the expression levels of the above genes.

### Statistical analysis

To determine the differences between each scaffold at each time point, one-way analysis of variance (ANOVA) and Student–Newman–Keuls post hoc tests were used for statistical analysis. Quantitative data are expressed as the mean ± standard deviation. A value of *P* < 0.05 was considered statistically significant. GraphPad Prism 7 software was used for all statistical analyses.

## Supplementary Information

**Additional file 1: Figure S1**. Elemental mapping images of SHA. SEM images (a1, a2). All elemental distribution images (b1, b2). Ca, P, O, Si distribution images (c1-f1, c2-f2), respectively. Macroporous surfaces (a1-f1) and microporous surfaces (a2-f2), respectively. **Figure S2**. Elemental mapping images of SMHA. SEM images (a1, a2). All elemental distribution images (b1, b2). Ca, P, O, Si, Mg distribution images (c1-g1, c2-g2), respectively. Macroporous surfaces (a1-g1) and microporous surfaces (a2-g2), respectively. **Figure S3**. Elemental mapping images of SSHA. SEM images (a1, a2). All elemental distribution images (b1, b2). Ca, P, O, Si, Sr distribution images (c1-g1, c2-g2), respectively. Macroporous surfaces (a1-g1) and microporous surfaces (a2-g2), respectively.

## Data Availability

The datasets used and/or analyzed during the current study are available from the corresponding author on reasonable request.

## Abbreviations

BTE: Bone tissue engineering
mHAws: Micron-sized HA whiskers
SHA: Silica-complexed mHAw scaffold
SMHA: Silica-complexed Mg-doped mHAw scaffold
SSHA: Silica-complexed Sr-doped mHAw scaffold
SMSHA: Silica-complexed Mg-Sr codoped mHAw scaffold
WCA: Water contact angle
SEM: Scanning electron microscopy
EDS: Energy dispersive spectroscopy
DMEM: Dulbecco’s modified Eagle’s medium
FBS: Fetal bovine serum
rBMSCs: Rat bone marrow-derived mesenchymal stem cells
BMSCs: Bone marrow mesenchymal stem cells
CCK-8: Cell counting Kit-8
RT-qPCR: Real-time quantitative polymerase chain reaction
COL1: Type I collagen
BMP2: Bone morphogenetic protein 2
Runx2: Runt-related transcription factor 2
ALP: Alkaline phosphatase
VEGF: Vascular endothelial growth factor
GAPDH: Glyceraldehyde 3-phosphate dehydrogenase
