# Supplementary material for: Characterization and in vitro assessment of three-dimensional extrusion Mg-Sr codoped SiO2-complexed porous microhydroxyapatite whisker scaffolds for biomedical engineering
Source: Biomed Eng Online. 2021 Nov 24;20:116. doi: 10.1186/s12938-021-00953-w (PMC8611959; doi:10.1186/s12938-021-00953-w)
Supplement: Supplementary file 1 — Additional file 1: Figure S1. Elemental mapping images of SHA. SEM images (a1, a2). All elemental distribution images (b1, b2). Ca, P, O, Si distribution images (c1-f1, c2-f2), respectively. Macroporous surfaces (a1-f1) and microporous surfaces (a2-f2), respectively. Figure S2. Elemental mapping images of SMHA. SEM images (a1, a2). All elemental distribution images (b1, b2). Ca, P, O, Si, Mg distribution images (c1-g1, c2-g2), respectively. Macroporous surfaces (a1-g1) and microporous surfaces (a2-g2), respectively. Figure S3. Elemental mapping images of SSHA. SEM images (a1, a2). All elemental distribution images (b1, b2). Ca, P, O, Si, Sr distribution images (c1-g1, c2-g2), respectively. Macroporous surfaces (a1-g1) and microporous surfaces (a2-g2), respectively. [file 12938_2021_953_MOESM1_ESM.doc]

Supporting Information

Characterization And in Vitro Assessment of Three-Dimensional Extrusion Mg-Sr Codoped SiO_2_-Complexed Porous Micro-Hydroxyapatite Whisker Scaffolds for Biomedical Engineering

Chengyong Li^1^, Tingting Yan^2^, Zhenkai Lou^1^, Zhimin Jiang^2^, Zhi Shi^1^, Qinghua Chen^2^, Zhiqiang Gong^1^, Bing Wang^1*^

1 Department of Orthopedics, First Affiliated Hospital of Kunming Medical University, Kunming Medical University, Kunming 650032, China; [lichengyonglcy666@163.com](mailto:lichengyonglcy666@163.com) (C.-Y.L.); [zhenkailou@163.com](mailto:zhenkailou@163.com) (Z.-K.L.); [2212128422@qq.com](mailto:2212128422@qq.com) (Z.S.); [493869301@qq.com](mailto:493869301@qq.com) (Z.-Q.G.); [drbingwang@163.com](mailto:drbingwang@163.com) (B.W.);

2 Faculty of Materials Science and Engineering, Kunming University of Science and Technology, Kunming 650093, China; [itty@foxmail.com](mailto:itty@foxmail.com) (T.-T.Y.); [2322102722@qq.com](mailto:2322102722@qq.com) (Z.-M.J.); [chenqinghua_yn1@163.com](mailto:chenqinghua_yn1@163.com) (Q.-H.C.);

* Corresponding author; Address correspondence to E-mail: [drbingwang@163.com](mailto:drbingwang@163.com)

The element distribution of SHA, SMHA and SSHA were shown in the EDS element mappings as follows (Figure S1-S3), respectively. No matter the macroporous furface or the microporous surface, each element were uniformly distributed on the corresponding scaffold.

**Figure S1.**


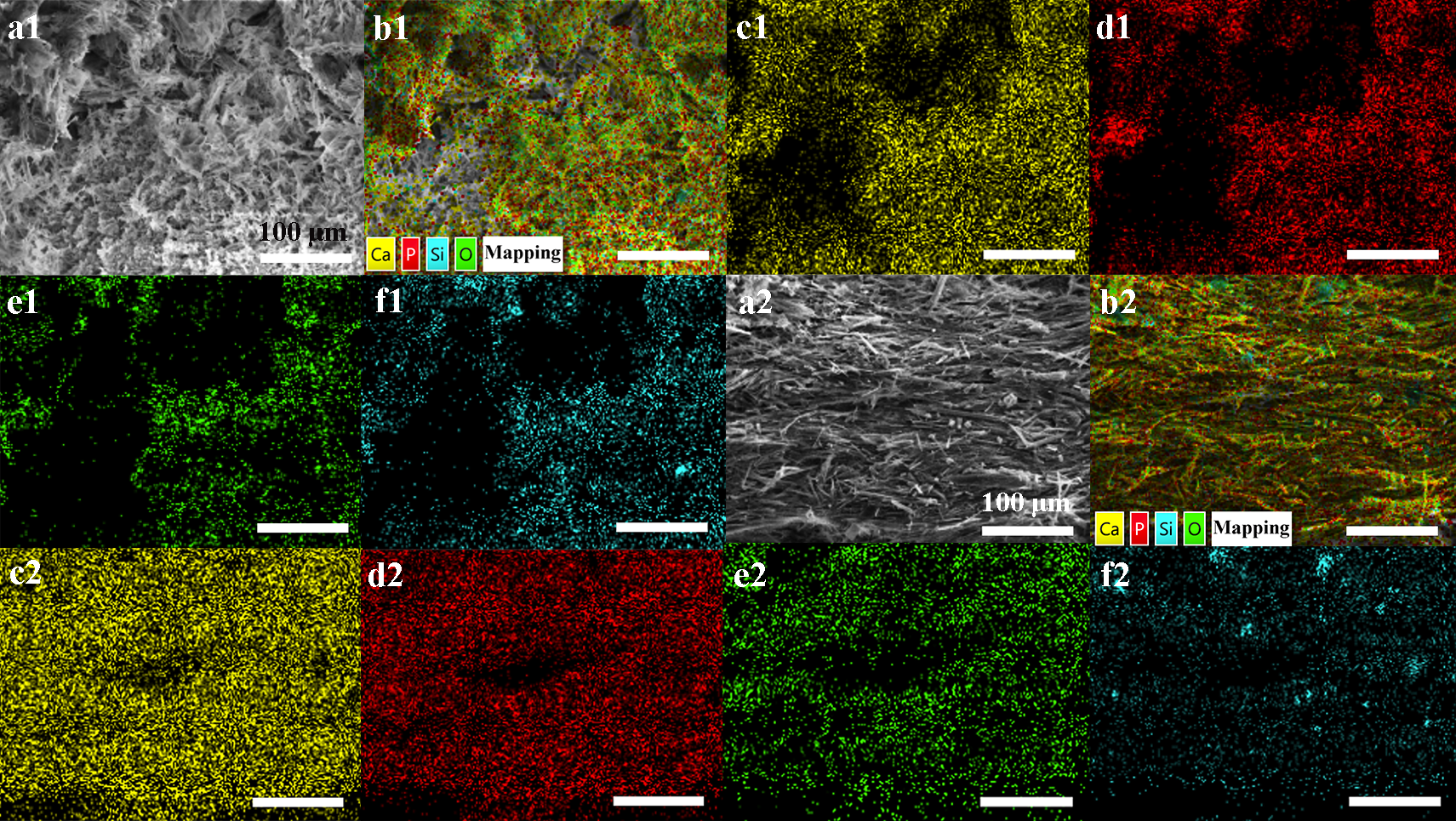


Figure S1. Elemental mapping images of SHA. SEM images (a1, a2). All elemental distribution images (b1, b2). Ca, P, O, Si distribution images (c1-f1, c2-f2), respectively. Macroporous surfaces (a1-f1) and microporous surfaces (a2-f2), respectively.

**Figure S2.**


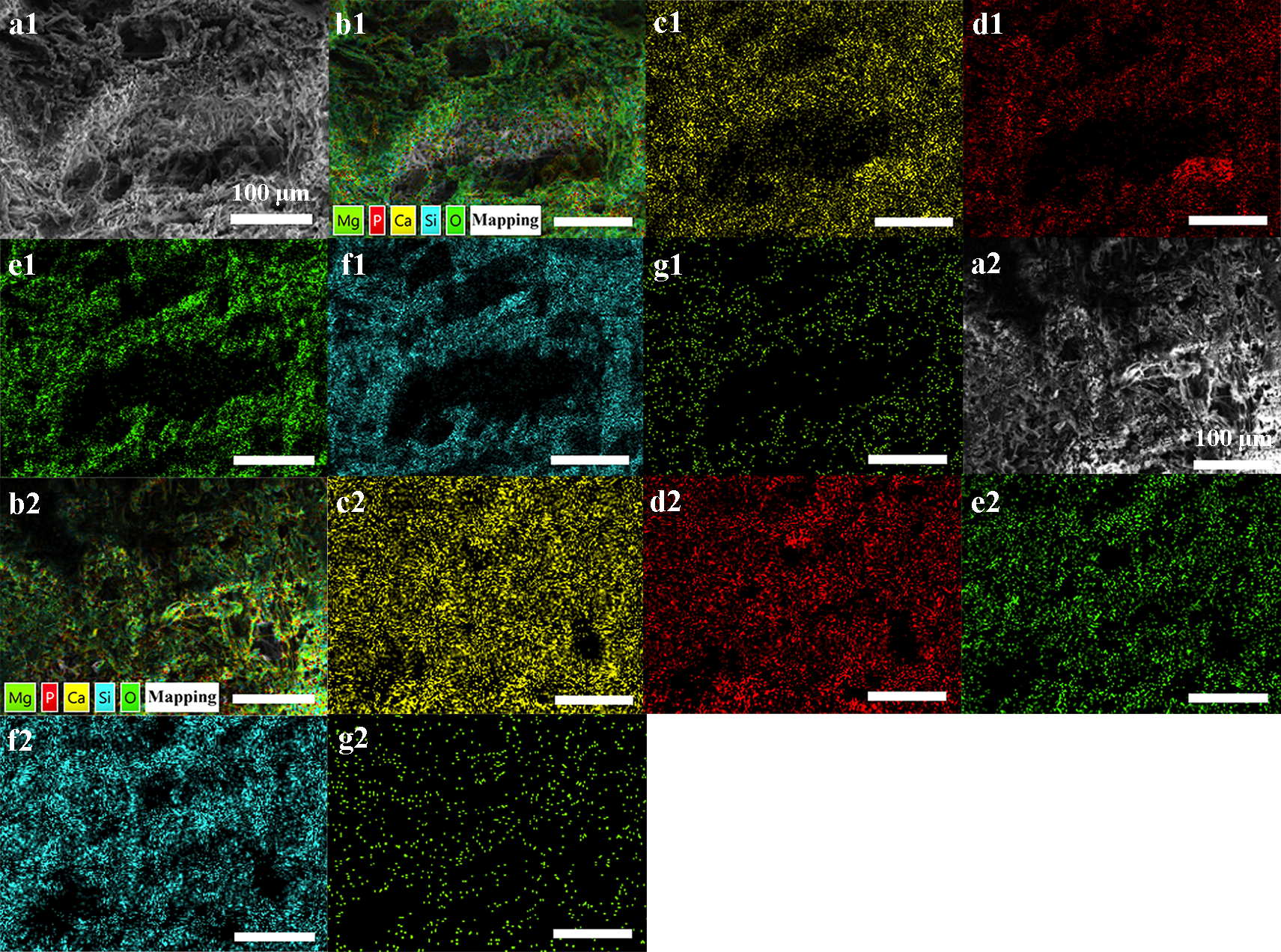


Figure S2. Elemental mapping images of SMHA. SEM images (a1, a2). All elemental distribution images (b1, b2). Ca, P, O, Si, Mg distribution images (c1-g1, c2-g2), respectively. Macroporous surfaces (a1-g1) and microporous surfaces (a2-g2), respectively.

**Figure S3.**


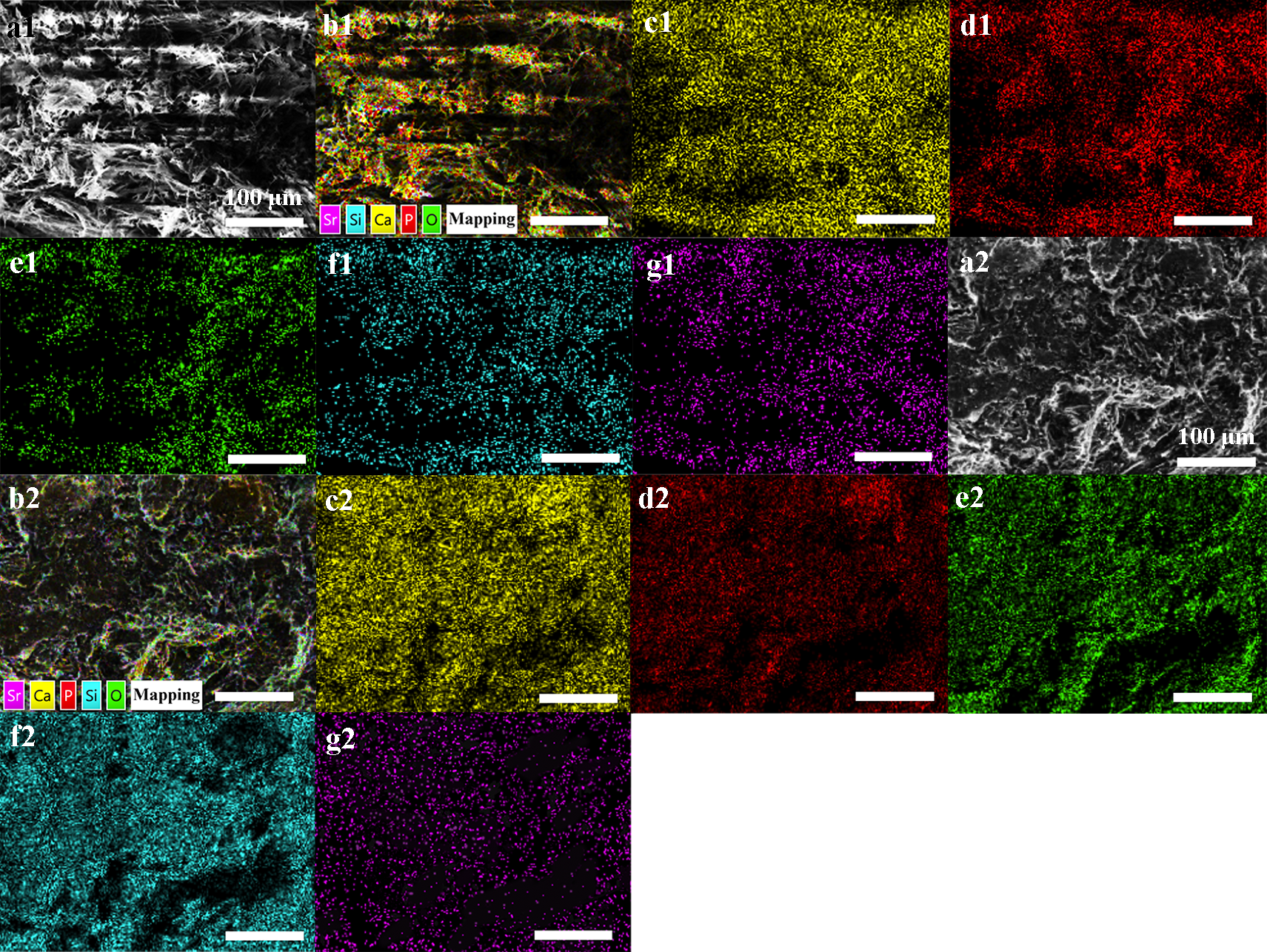


Figure S3. Elemental mapping images of SSHA. SEM images (a1, a2). All elemental distribution images (b1, b2). Ca, P, O, Si, Sr distribution images (c1-g1, c2-g2), respectively. Macroporous surfaces (a1-g1) and microporous surfaces (a2-g2), respectively.
